# Safety and neurological assessments after autologous transplantation of bone marrow mesenchymal stem cells in subjects with chronic spinal cord injury

**DOI:** 10.1186/scrt516

**Published:** 2014-11-17

**Authors:** Marcus Vinícius Pinheiro Mendonça, Ticiana Ferreira Larocca, Bruno Solano de Freitas Souza, Cristiane Flora Villarreal, Luiz Flávio Maia Silva, André Costa Matos, Marco Antonio Novaes, Cláudia Maria Pinheiro Bahia, Ana Carine de Oliveira Melo Martinez, Carla Martins Kaneto, Sissi Brandão Carneiro Furtado, Geraldo Pedral Sampaio, Milena Botelho Pereira Soares, Ricardo Ribeiro dos Santos

**Affiliations:** Hospital Espanhol, Av. Sete de Setembro, 4161 Barra, Salvador, BA 40140-110 Brazil; Centro de Pesquisas Gonçalo Moniz, Fundação Oswaldo Cruz, R. Waldemar Falcão, 121, Candeal, Salvador, BA 40296-710 Brazil; Centro de Biotecnologia e Terapia Celular, Hospital São Rafael, Avenida São Rafael, 2152, São Marcos, Salvador, BA 41253-190 Brazil; Universidade Federal da Bahia, Salvador, BA Brazil; Centro Universitário Estácio da Bahia – FIB, Estãcio-FIB - R. Xingu, 179 - Jardim Atalaia, STEIP, Salvador, BA 41770-130 Brazil

## Abstract

**Introduction:**

The administration of stem cells holds promise as a potential therapy for spinal cord injury (SCI). Mesenchymal stem cells have advantages for clinical applications, since they can be easily obtained, are suitable for autologous transplantation and have been previously shown to induce regeneration of the spinal cord in experimental settings. Here we evaluated the feasibility, safety and potential efficacy of autologous transplantation of mesenchymal stem cells in subjects with chronic complete SCI.

**Method:**

We conducted a phase I, non-controlled study in 14 subjects of both genders aging between 18 to 65 years, with chronic traumatic SCI (>6 months), at thoracic or lumbar levels, classified as American Spinal Injury Association (ASIA) A - complete injury. Baseline somatosensory evoked potentials (SSEP), spinal magnetic resonance imaging (MRI) and urodynamics were assessed before and after treatment. Pain rating was performed using the McGill Pain Questionnaire and a visual analogue score scale. Bone marrow-derived mesenchymal stem cells were cultured and characterized by flow cytometry, cell differentiation assays and G-band karyotyping. Mesenchymal stem cells were injected directly into the lesion following laminectomy and durotomy.

**Results:**

Cell transplantation was an overall safe and well-tolerated procedure. All subjects displayed variable improvements in tactile sensitivity and eight subjects developed lower limbs motor functional gains, principally in the hip flexors. Seven subjects presented sacral sparing and improved American Spinal Injury Association impairment scale (AIS) grades to B or C – incomplete injury. Nine subjects had improvements in urologic function. One subject presented changes in SSEP 3 and 6 months after mesenchymal stem cells transplantation. Statistically significant correlations between the improvements in neurological function and both injury size and level were found.

**Conclusion:**

Intralesional transplantation of autologous mesenchymal stem cells in subjects with chronic, complete spinal cord injury is safe, feasible, and may promote neurological improvements.

**Trial registration:**

ClinicalTrials.gov NCT01325103 – Registered 28 March 2011

## Introduction

Severe spinal cord injury (SCI), leading to chronic paraplegia, is considered an irreversible condition for which there is currently no effective clinical therapy [1]. The main goal of stem cell therapy in SCI is to repair the damaged neuronal tissue. In experimental models of SCI, stem cell transplantation has been used as a new strategy to overcome physical disability and promote neurological improvements. Several studies have indicated a beneficial role for transplantation of different cell types, including bone marrow cells, neural progenitor cells and olfactory ensheathing cells, into the injured spinal cord [2–6]. Another approach that has been tested is the use of oligodendrocyte progenitor cells to promote oligodendrogenesis after SCI, which led to remyelinization and motor function improvement [7].

Results in preclinical studies have encouraged researchers to undertake clinical trials in SCI with cell therapy, mainly with mesenchymal stem cells (MSCs), Schwann cells and human olfactory ensheathing glia (reviewed in [8]). Special attention has been given to MSCs, which are shown to promote neural repair and regeneration of damaged areas in different experimental models [9–12]. MSCs exhibit a broad degree of plasticity, with the ability to differentiate not only into multiple mesodermal lines, including bone, fat, muscle, liver and cartilage cells [13–17], but also into cells expressing neural and glial lineage markers [18, 19]. In addition, it has been reported that MSCs secrete cytokines and growth factors that promote autocrine and paracrine effects, such as immunosuppression, inhibition of gliosis and apoptosis, enhanced angiogenesis, axon sorting and myelination [20, 21]. In previous studies, we have established a protocol for MSC intralesional transplantation in dogs and cats with naturally acquired SCI [22, 23].

Clinical use of MSCs presents several advantages, including easy isolation from bone marrow aspirates and large-scale expansion in culture, allowing autologous transplantations [18, 22]. Moreover, to date, *in vivo* tumor development has not been reported, either in experimental models or in clinical trials in which MSCs were used to treat SCI [23–25]. We conducted a clinical trial to evaluate the safety and potential efficacy of autologous bone marrow MSC transplantation in subjects with chronic thoracic and lumbar SCI caused by trauma.

## Methods

### Ethics statement

This phase I, nonrandomized, uncontrolled, prospective, open-label study was approved by the Brazilian National Council of Ethics in Research (CONEP) [registration number 14942, SIPAR/MS: 25000.112358/2008–71] and was registered on the National Institute of Health database [ClinicalTrials.gov:NCT01325103] [26]. Ethical guideline provisions from the Helsinki Declaration were followed. Written consent for participation and for publication was obtained from the subjects.

### Objectives and outcomes

The main objective was to evaluate the safety of autologous bone marrow MSC transplantation in subjects with chronic traumatic SCI. The secondary objective was to assess potential efficacy, through neurological improvements in sensory and motor assessment (measured by American Spinal Injury Association (ASIA) scores), pain scores, urodynamic studies and evoked potential studies.

The safety outcome was defined as deleterious modifications on resonance magnetic imaging, as well as possible side effects and adverse events related to the protocol procedures. The primary outcome was defined as neurological improvements on ASIA scores (light touch, pin prick and motor power), as described previously [27]. The secondary outcomes were defined as improvements on pain scores, urodynamic study and evoked potential somatosensory test. The subjects were followed up to 6 months postoperatively. All data were collected personally by the same researchers, through specific forms and validated questionnaires, in order to enhance the quality of measurements and results.

### Subject selection

Fourteen subjects who fit the following inclusion criteria were recruited: traumatic SCI at the thoracic or lumbar level, American Spinal Injury Association impairment scale (AIS) grade A, age ≥18 and ≤65 years, and previous surgical intervention for spinal cord decompression and stabilization. The sample size was determined as the minimal number of subjects necessary to evaluate the procedure safety. All data were collected at Hospital São Rafael and Hospital Espanhol in Salvador, Bahia, Brazil.

Exclusion criteria were anatomical transection of the spinal cord, open SCI (for example, stabbing, gunshot wound), concurrent infectious disease, terminal illness, neurodegenerative disorders, primary hematological disorders, osteopathies, coagulopathies, hepatic dysfunction, other clinical complications that could contraindicate the surgery, use of metallic implants that contraindicate magnetic resonance imaging (MRI) and participation in other clinical trials.

Participants and researchers, those administering the interventions and those assessing the outcomes, were not blinded to study condition assignment.

### Isolation of bone marrow cells and mesenchymal stem cell culture

Before the procedure, subjects were assessed for hematology, blood biochemistry, urine microbiology and screening for HIV, human T-cell lymphotropic virus, Chagas disease, and hepatitis B and C status. Bone marrow aspiration was performed in an outpatient surgery center. Subjects were sedated prior to the procedure and monitored by an anesthesiologist. After local anesthesia using 2% lidocaine, approximately 60 ml bone marrow were aspirated from the anterior and posterior iliac crest using a specific needle of bone marrow puncture of adjustable length (1.0 to 4.8 cm) and 15 gauge (Carefusion, San Diego, CA, USA) and were collected in 20 ml syringes containing 1 ml of 5,000 UI heparin (Cristália, Itapira, Brazil). The collection of bone marrow cells was performed by a hematologist at Hospital São Rafael in Salvador, Bahia, Brazil.

Cell separation and culture procedures were performed in a certified current good manufacturing practice facility using standardized procedures. The mononuclear cell fraction was separated from the bone marrow by centrifugation under Ficoll-Hypaque gradient (GE Healthcare Life Sciences, Little Chalfont, UK) at 2,000 rpm without a break, at room temperature, for 30 minutes. After three washes in saline solution, cells were plated in culture flasks (TPP, St. Louis, MO, USA) at a density of 1.3 × 10^5^ cells/cm^2^. The cells were cultured in minimum essential Eagle’s medium with alpha modifications supplemented with l-glutamine (2 mM/l), 1% gentamycin, 2.4 g/l Hepes, 2 g/l sodium bicarbonate (all from GIBCO, Grand Island, NY, USA) and enriched with 15% fetal bovine serum (Hyclone/Thermo Scientific, Logan, UT, USA). One-half of the medium was exchanged every 3 days. Once the cells achieved 80 to 90% confluence, they were dissociated with 0.25% porcine trypsin/0.53 mM ethylenedinitrilotetraacetic acid (Invitrogen/Life Technologies, Grand Island, NY, USA) and replated at a density of 8,000 cells/cm^2^. MSCs were expanded for approximately 4 weeks until adequate transplantation numbers were achieved. Confluent autologous MSCs at passages 3 to 5 were resuspended in saline solution containing 20% human serum albumin (CSL Behring, King of Prussia, PA, USA). MSC suspensions (1 × 10^7^ cells/ml) were transferred into 1 ml syringes for local injection in subjects. Before transplantation, the cells were characterized by flow cytometry analysis, differentiation assays and G-band karyotype analysis and tested for sterility.

### Cell differentiation assays

To assess the differentiation potential of MSCs, 40,000 cells/ml were cultured in 24-well plates (Greiner, Monroe, NC, USA) with coverslips for performing morphological studies. When differentiation was initiated, the entire culture medium was removed and replaced by induction medium (specific for osteoblasts, adipocytes and chondrocytes) or only minimum essential Eagle’s medium with alpha modifications supplemented with 7.5% fetal bovine serum as control. Commercial kits were used for adipogenic, chondrogenic and osteogenic induction media following the manufacturer’s recommendations (StemPro adipogenesis Differentiation Kit, StemPro Chondrogenesis Differentiation Kit and StemPro Osteogenesis Differentiation Kit; GIBCO). MSCs were continuously evaluated by phase-contrast microscopy during the differentiation process. Histochemical staining was also used for cell morphology evaluation during the differentiation process. MSCs differentiated into adipocytes and their controls were stained with Sudan II for visualization of lipid inclusions. The cells that differentiated into chondrocytes and their controls were stained with Alcian Blue for staining of proteoglycan deposits. Cells that differentiated into osteoblasts and their controls were stained with Von Kossa for visualization of mineralized matrix. Morphological evaluation of cells was performed using an AX70 optical microscope coupled to a digital camera for imaging capture (Olympus, Tokyo, Japan).

### Flow cytometry analysis

For immunophenotyping, adherent MSCs were detached with 0.25% porcine trypsin solution (Invitrogen), washed with saline and incubated at 4°C for 30 minutes with the following antibodies: fluorescein isothiocyanate anti-human CD31, phycoerythrin anti-human CD34, phycoerythrin anti-human CD117, phycoerythrin anti-human CD14, allophycocyanin anti-human CD90, phycoerythrin anti-human CD79a, allophycocyanin anti-human CD44, peridinin chlorophyll protein anti-human CD45, fluorescein isothiocyanate anti-human CD19 (all from BD-Pharmingen, San Diego, CA, USA) and fluorescein isothiocyanate anti-human CD105 (R&D Systems, Minneapolis, MN, USA). The acquisition and analysis were carried out using a LSR Fortessa cytometer with the FACSDiva software (Becton Dickinson, San Jose, CA, USA). At least 10,000 events were collected.

### Cytogenetic evaluation

Cytogenetic analysis was performed in MSC samples from all subjects, during every passage. All of the analyses were performed prior to transplantation in order to detect possible chromosomal mutations induced by culture conditions. MSCs were treated with 16 μg/ml colchicine (Cultilab, Campinas, Brazil) for a period of 6 hours for cell cycle arrest at metaphase. Cells were trypsinized, resuspended, centrifuged, exposed to hypotonic solution of 0.075 M KCl, placed in a water bath at 37°C for 30 minutes and fixed with Carnoy’s solution 3:1 (acetic acid:methanol).

Cytogenetic analysis was performed by the GTG banding technique. G bandings for the prepared slides were aged at 60°C overnight and subjected to treatment with a solution of 0.1% trypsin/phosphate-buffered saline and subsequently stained with Giemsa solution/phosphate-buffered saline. Twenty cells were analyzed for each passage. The analysis of these cells was performed using a BX61 microscope (Olympus) and images were captured using a digital imaging system (Applied Spectral Imaging, Carlsbad, CA, USA) coupled to the microscope. Results were interpreted according to the International System for Human Cytogenetic Nomenclature classification.

### Cell transplantation and subject follow-up

Subjects in the prone position under general anesthesia underwent a midline incision comprised of two levels above and two levels below the injury. Following laminectomy and decompression of the injured spinal canal segment, the dura mater was opened under the microscope to visualize the injured spinal segment. A fixed cell number (5 × 10^6^ cells/cm^3^) was injected per lesion volume. The estimation of lesion volume was performed by MRI analysis, using the ellipsoid formula, as described previously [28].

The injection was performed over a period of 5 minutes. Punctures were made in the cardinal directions of the injured area and one level above and below, before closing the dura mater. The whole procedure was performed only once, by a neurosurgeon, at Hospital Espanhol in Salvador, Bahia, Brazil.

As an incentive strategy, in order to increase compliance, subjects underwent rehabilitation for 6 months after the surgery, five times a week, for 4 hours a day during the first 2 months and 2 hours a day in the subsequent months. Regular clinical and neurological assessments were performed for at least 6 months. At each follow-up, a complete clinical assessment, neurological evaluation and AIS scale assessment were conducted. A urodynamic study, somatosensory evoked potentials (SSEP) and MRI of the spine were carried out in months 3 and 6 of follow-up.

### Clinical pain measures

All pain measurements were performed in a quiet room with the temperature maintained between 21 and 23°C. At the time of testing, subjects rated their present pain using an unanchored visual analogue score (VAS). Data from the VAS scale were presented in millimeters. Next, subjects were asked to indicate where they were currently experiencing chronic pain by shading in the areas on a drawing of the dorsal and frontal views of the human body. Following this, subjects were also asked to fill in a standard Brazilian-Portuguese language version of the McGill Pain Questionnaire [29], the results of which were then quantified using the pain rating index [30].

### Sensory assessment

Test sites were identified based on anatomical landmarks to ensure that the same site could be accurately located in subsequent sessions. For each participant, a starting stimulation site was selected based on each individual’s level of injury, as determined by the ASIA scale. The starting site was defined as areas at least four dermatomes above the neurological level of injury, where sensation was expected to be within normal limits. The mechanical stimulation response was measured with calibrated von Frey filaments (Touch Test Sensory Evaluator; Stoelting, Wood Dale, IL, USA). Each subject was instructed to close his or her eyes during this portion of the testing and respond with 'yes’ if he or she could feel the test stimulus when it was delivered or with 'no’ if he or she could not feel the stimulus. For each trial, the monofilament (10 g) was applied perpendicular to the skin surface and, once the filament was fully bent, was held in place for approximately 1 second before being lifted off the skin. Following a positive response, the next area below was stimulated.

### Urodynamics

The urodynamic study was performed prior to and 3 to 6 months after the transplantation of MSCs. The following parameters were measured at cystometry: maximum bladder capacity, compliance, bladder sensation, presence of detrusor overactivity and presence of urinary incontinence.

Compliance was measured when the bladder showed filling ability greater than 200 ml in the absence of detrusor overactivity. Bladder sensation was marked as absent, partially preserved or completely preserved, as described previously [31].

Those subjects with micturition were also evaluated with the pressure-flow study. For the urodynamic study, we used Dynamed Pro-Life Technology, São Paulo, Sp, Brazil dynapack mpx 816 equipment. For cystometry, two plastic urethral probes were inserted into the bladder (6 Fr to measure intravesical pressure and 8 Fr for filling). A 10 Fr rectal probe were inserted for measuring intraabdominal pressure. The filling was done with distilled water at room temperature at a rate of 40 ml/minute.

### Somatosensory evoked potentials

The SSEP were evaluated before and 3 and 6 months after MSC transplantation. The examinations were performed using Neuropack M1 (Nihon Kohden, Tokyo, Japan) four-channel equipment, with tibial nerve stimulation for evaluation of the lower limbs, registration in the popliteal fossa, lumbar (L2/3) and scalp (Cz′ to Fpz) regions, and median nerve stimulation for evaluation of the upper limbs, with registration at Erb’s point, cervical C5 and scalp (C3′ to C4′). In the event that some potentials were obtained, the evaluation was replicated at least three times to assess consistency.

### Statistical analyses

The individual was the smallest unit analyzed to assess intervention effects (same from the unit of assignment). Missing data were not used.

A paired *t* test was used to analyze ASIA scores for light touch and pin prick, before and 6 months after transplantation. The nonparametric Wilcoxon signed-rank test was employed to test the existence of a statistically significant difference between ASIA motor scores before and 6 months after transplantation. Pearson correlation analysis was performed to evaluate possible correlations between ASIA scores (light touch, pin prick and motor power) and lesion characteristics (volume, level and time of lesion). Statistical analyses were performed using Prism Software (version 3.0; GraphPad Software, San Diego, CA, USA). Differences were considered significant if *P* ≤ 0.05.

## Results

### Subjects

Regarding the enrollment, 555 participants were screened for eligibility, 205 were found to be eligible and 14 were enrolled (10 males), following the order of first contact date. Fourteen subjects were assigned to the study condition and were submitted to bone marrow aspiration and MSC transplantation. Of the 14 subjects assigned, two were considered to have lost follow-up; one due to leg and urethral injuries unrelated to the study protocol, and the other due to lack of compliance with the assessments. These two subjects, considered noncompliers, were excluded from the main analysis.

Subjects had chronic traumatic SCI with a mean duration of approximately 61.7 months (ranging from 18 to 180 months). Four subjects were female and 10 were male, with mean age of 35.7 ± 9.9 years, ranging from 23 to 61. All were classified as ASIA grade A and had injuries in the lumbar or thoracic segments of the spinal cord. The lesion volumes were estimated by MRI analysis, with a mean of approximately 3.66 cm^3^, ranging from 0.77 to 10.44 cm^3^. The demographic, clinical, radiological and neurological features of the subjects are presented in Table 1.

**Table 1:** **Demographic, clinical and neurological features of the subjects**

Subject	Months post SCI	SCI level	AIS grade	Lesion (cm ^3^ )
1	101	T12	A	2.7
2	42	T5	A	1.0
3	36	L1	A	5.4
4	25	T12	A	5.0
5	18	T5	A	1.0
6	29	T7	A	2.7
7	91	T5	A	4.0
8	153	T12	A	4.0
9	180	T12	A	0.8
10	27	T5	A	0.9
11	52	T12	A	7.5
12	19	T7	A	10.4
13	66	T7	A	3.0
14	26	T12	A	3.0

AIS, American Spinal Injury Association impairment scale; SCI, spinal cord injury.

### Study protocol deviations

Urodynamic data were not properly collected due to recurrent urinary infection in some of the subjects, who could not be submitted to the urodynamic study in the correct timeframe.

### Mesenchymal stem cell characterization

**Figure 1:**
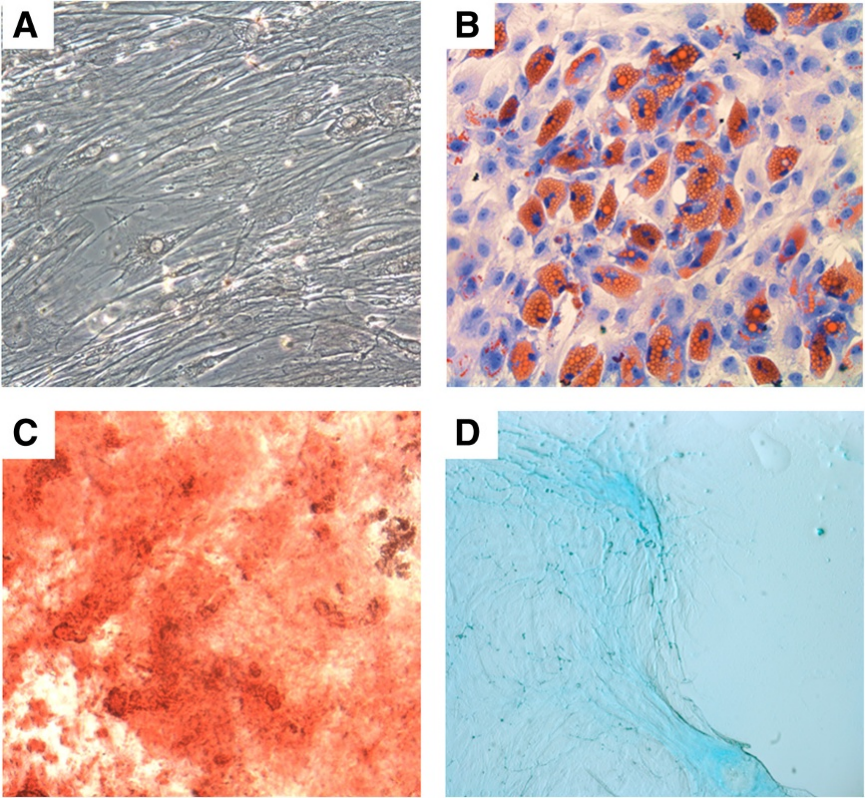
**Cell differentiation assays.** Mesenchymal stem cells **(A)** were cultured in the presence of adipogenic **(B)**, osteogenic **(C)** and chondrogenic **(D)** differentiation media. Cultures were stained with Sudan II, von Kossa and Alcian blue, respectively. Representative images obtained from mesenchymal stem cell cultures from one patient. Magnification = 400×.

**Figure 2:**
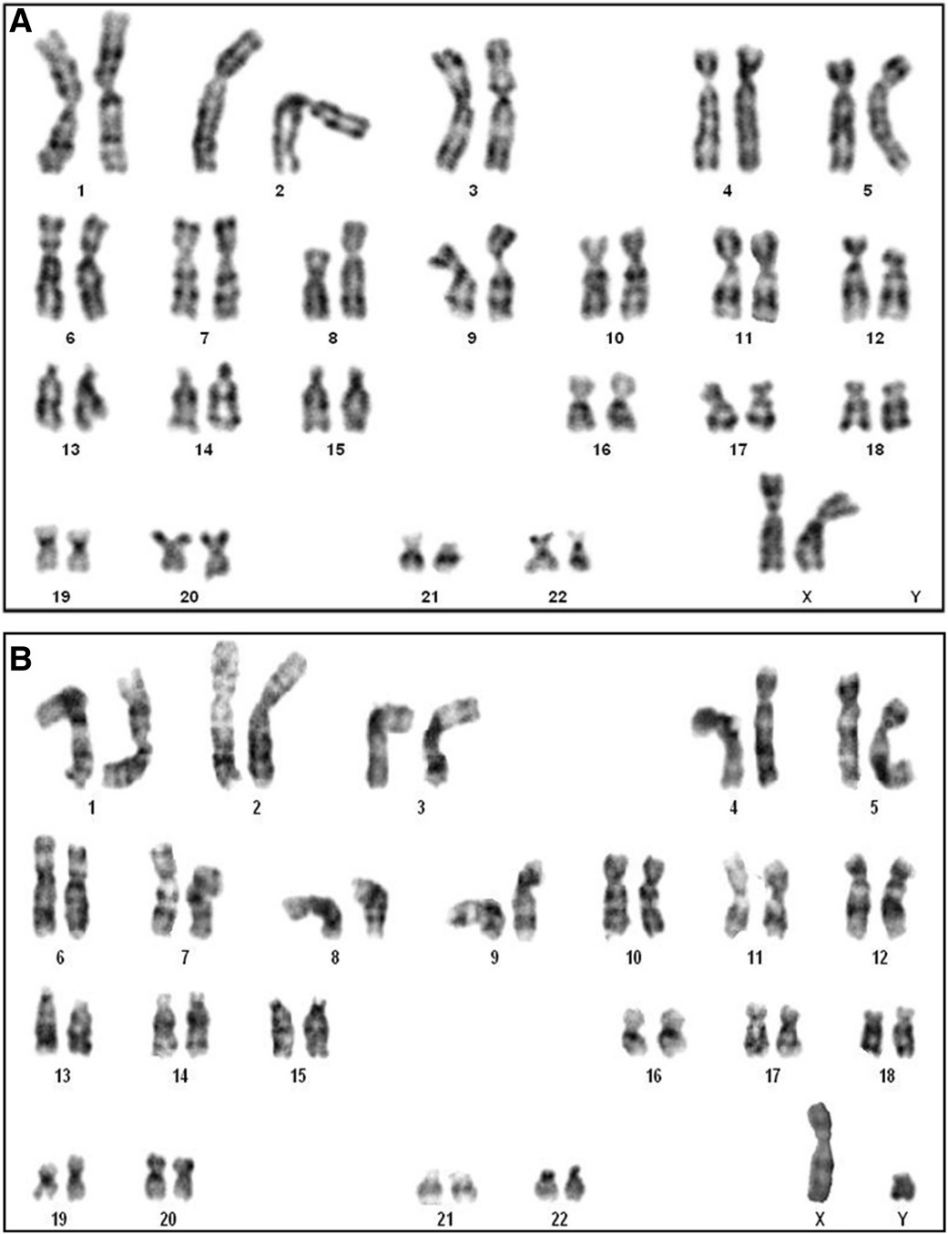
**Karyotype of bone marrow-derived mesenchymal stem cells by GTG banding. (A)** Patient 7: 46,XX [20]. **(B)** Subject 10: 46,XY.

Cultured bone marrow-derived cells presented a fibroblast-like morphology and showed 97.4 ± 3.1% cells positive for CD105, 97.5 ± 2.8% cells positive for CD73 and 96.6 ± 3.5% cells positive for CD90. Moreover, MSC cultures presented 2.0 ± 2.1% cells positive for CD45, 2.3 ± 2.6% cells positive for CD14, 2.4 ± 3.0% cells positive for CD79 and 2.3 ± 2.5% cells positive for CD34. The MSCs were successfully induced to differentiate into chondrocytes, osteocytes and adipocytes (Figure 1). Additionally, no chromosomal aberrancies were detected in G-band karyotype analysis in the passages used for transplantation (Figure 2).

### Adverse effects

Transplantation of bone marrow-derived MSCs was an overall safe procedure. All of the subjects were discharged within 48 hours after surgery. The most frequent postoperative symptom was low-intensity pain at the incision site, which was responsive to regular analgesics. One subject developed a postoperatory complication, evolving a cerebrospinal fluid leak that was treated by an additional surgical procedure. None of the subjects had fever, infection or meningitis. Subjects underwent a program of rehabilitation beginning 1 week after the surgical procedure, which was well tolerated.

### Clinical assessments

**Figure 3:**
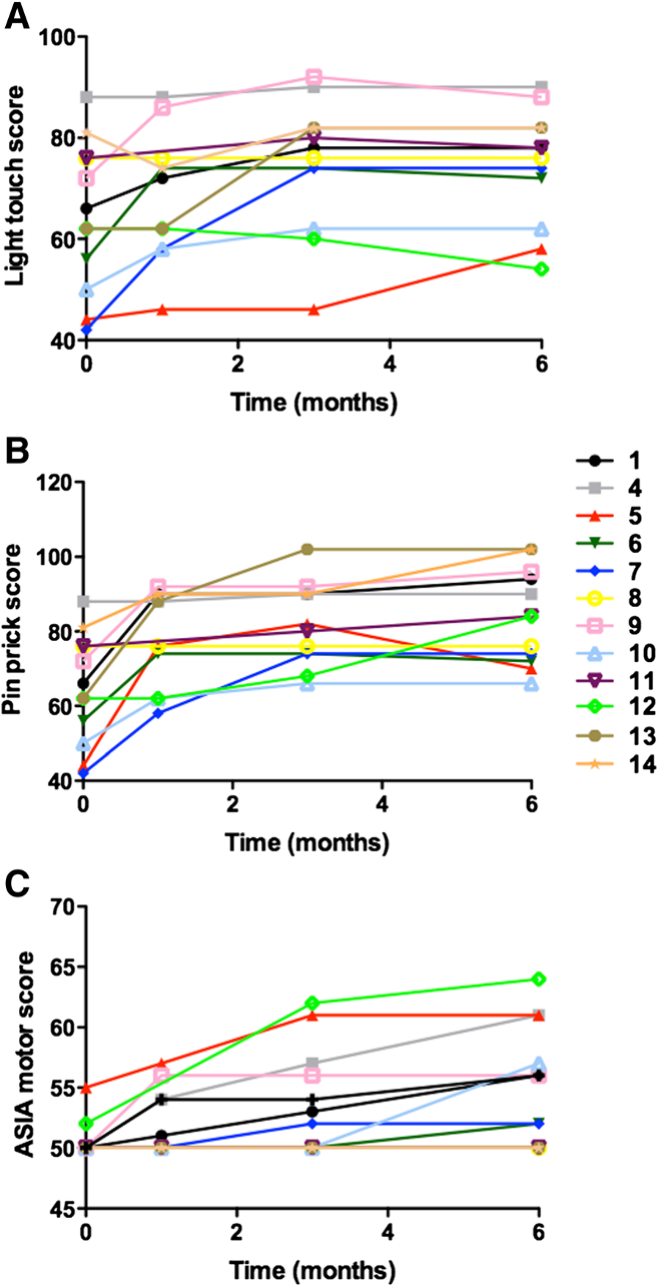
**American Spinal Injury Association scores before and during follow up.** Sensory scores evaluated by the light touch **(A)** and pin prick **(B)**. American Spinal Injury Association (ASIA) motor score **(C)**.

**Figure 4:**
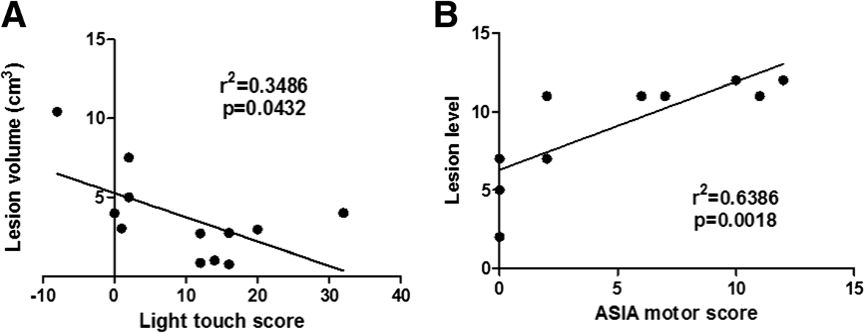
**Analyses of correlation between lesion characteristics and American Spinal Injury Association scores. (A)** Pearson correlation analysis of lesion volume (cm^3^) and light touch American Spinal Injury Association (ASIA) score. **(B)** Pearson correlation analysis of lesion level and ASIA lower limb motor improvement. Lesion level was consecutively numbered starting from T1.

Neurological evaluation revealed variable improvements in sensitivity below the lesion level following treatment, as assessed by light touch and pin prick (Figure 3A,B). Light touch and pin prick ASIA sensitivity score analysis, prior to and 6 months after transplantation, demonstrated a statistically significant improvement (*P* <0.01 and *P* <0.001, respectively; paired *t* test). All subjects showed some degree of sensitivity gain in response to mechanical stimulation when measured with von Frey monofilaments (50% had significant improvements, comparing with the presurgical profile). Subject 1 demonstrated sensitivity recovery in all dermatomes (Table 2). Major sensitivity gains were measured in the first 3 months after MSC transplantation (Figure 3A,B; Table 2). The Pearson coefficient showed an inverse correlation between light touch gain 6 months after transplantation and lesion volume (*r*^2^ = 0.3486; Figure 4A). No significant correlations were observed when light touch or pin prick sensitivity gains were analyzed in relation to level or time of lesion.

**Table 2:** **Response to mechanical stimulation**

Subject number	Dermatomes with positive response to von Frey stimulation ^a^			
	Before treatment	Months after treatment		
		1	3	6
1	T11/T11	L1/L1	ALL	ALL
4	L2/L2	L2/L3	L3/S2	S2/S2
5	T4/T4	T4/T4	T5/T4	T5/T4
6	T5/T4	T5/T5	T6/T5	T6/T6
7	T4/T3	T5/T4	T5/T4	T5/T4
8	T11/T11	T12/T12	T12/T12	T12/T12
9	T12/L1	L3/L3	L3/L3	L3/L3
10	T5/T5	T5/T6	T6/T6	T7/T6
11	T11/L1	L1/L2	L1/L2	L2/L2
12	T4/T4	T5/T5	T6/T5	T6/T5
13	T5/T4	T7/T6	T8/T6	T7/T6
14	T11/T10	T12/T11	L1/T11	L5-S1/T12

^a^Side of the body (right/left).

Improvements in lower limb motor function were observed in eight subjects (Figure 3C). Two subjects presented gains in hip flexor function, three subjects had gains in knee extension movement and three subjects demonstrated gains in ankle dorsiflexion. The most frequent alteration was the increased motor power observed in hip-related muscle groups. A statistically significant improvement in ASIA motor scores was observed when comparing before and 6 months after transplantation (*P* <0.05; Wilcoxon signed-rank test). The Pearson coefficient demonstrated a direct correlation between motor gain and lesion level (*r*^2^ = 0.6386) 6 months after transplantation (Figure 4B). No significant correlations were seen when motor gains were analyzed in relation to volume or time of lesion.

Additionally, seven subjects presented sacral sparing after MSC transplantation, having recovered anal sensation. Of these, six subjects had changes in the AIS grade to grade B and one subject to grade C (Table 3).

**Table 3:** **Summary of American Spinal Injury Association impairment scale grades**

Subject	Baseline	3 months	6 months
1	A	A	B
4	A	A	A
5	A	A	A
6	A	A	A
7	A	A	A
8	A	B	B
9	A	B	B
10	A	A	B
11	A	A	C
12	A	A	A
13	A	B	B
14	A	B	B

### Urological study

During the complete treatment and evaluation period, renal function did not deteriorate in any of the subjects. The maximum cystometric capacity changed from 203 ± 113 ml to 242 ± 146 ml, without statistical significance. Bladder compliance improved significantly from 14.7 ± 8.2 to 25.4 ± 15.9 ml/cmH_2_O (*P* =0.02). Five subjects who presented bladder sensation previously classified as absent improved to present a reduced sensation. Ten subjects began this study presenting detrusor overactivity during bladder filling; however, following MSC transplantation, four of these subjects increased the filling volume while three subjects decreased the filling volume up to the first involuntary contraction. Throughout the study, all subjects remained with urinary incontinence and in need of intermittent urinary catheterization.

### Radiological evaluation

Nuclear magnetic resonance images obtained before MSC transplantation revealed the presence of spinal cord cavities in eight subjects and syrinx in two subjects. Atrophy cord areas and gliosis, as well as findings associated with the primary surgery (that is, epidural fibrosis, soft tissue), were found in all subjects. MRI analysis 3 and 6 months after MSC transplantation revealed no alterations in hyperintense signals, extension of cavities or appearance of new gliosis areas. Moreover, no signs of ectopic tissue formation were observed during the follow-up.

### Pain assessment

Three subjects presented transient worsening of neuropathic pain, which was pharmacologically controlled. Three subjects ameliorated the neuropathic pain 3 months post treatment, with significant reduction of pharmacological therapy dependency.

Clinical pain measurements, administered through the VAS and pain rating index from the McGill Pain Questionnaire, were assessed prior to and 1, 3 and 6 months following MSC transplantation (Table 4). Although a reduction in the VAS and pain rating index was observed in 67% of subjects 6 months after surgery, there were no statistically significant differences observed when subjects were compared at 0 and 6 months after MSC transplantation (*P* = 0.0538 and *P* = 0.1211, respectively; paired *t* test).

**Table 4:** **Clinical pain measures**

Subject	VAS				PRI			
	Before	Months after treatment			Before	Months after treatment		
		1	3	6		1	3	6
1	3.0	0	0	0	6.6	0	0	0
4	5.0	5.0	6.0	6.0	17.0	19.3	16.5	20.5
5	4.0	6.0	6.0	0	8.5	14.5	11.0	0
6	6.0	6.0	7.0	3.0	17.2	25.0	30.3	13
7	1.0	0	0	0	7.0	0	0	0
8	7.0	7.0	7.0	7.0	26.4	24.0	26.4	31.2
9	10.0	8.0	8.0	8.0	33.7	29.5	24.3	22.2
10	5.0	3.0	7.0	7.0	11.3	10.6	24.8	18.1
11	8.0	8.0	7.0	0	40.5	37.0	44.9	0
12	6.5	3.0	3.0	3.0	23.8	8.4	15.6	10.9
13	6.0	0	7.0	8.5	28.5	0	21.1	41.1
14	10.0	10.0	9.0	7.0	46.0	43.0	27.6	28.8

PRI: pain rating index from the McGill Pain Questionnaire; VAS, visual analogue score for pain in millimeters.

### Somatosensory evoked potential

Subjects were assessed for SSEP before, 3 and 6 months after MSC transplantation. Lower sensory nerve electrical stimulation-mediated SSEP were not evoked in any subject prior to transplantation. Only one subject (Subject 14) presented an improved SSEP response in the left side 3 months after MSC transplantation, which was maintained until the follow-up conclusion. Despite the fact that the SSEP recorded in this subject presented a low amplitude, they were consistent and demonstrated the cortical response in P37/N45 on the left side.

## Discussion

A growing body of evidence, both experimental as well as clinical, has demonstrated that spontaneous plasticity events may occur within the post-traumatically injured spinal cord, through mechanisms including alterations in the properties of spared neuronal circuits, intact or injured axon collateral sprouting and synaptic rearrangements, as reviewed by Onifer and colleagues [32]. Nonetheless, although there is an abundance of studies describing the natural history of neurologic functional gains during the first year post SCI, there is a lack of data regarding the degree of neurological recovery that may naturally occur during prolonged follow-up investigation. A previous study described that 16.9% of subjects with complete SCI improved motor levels between years 1 and 5 after SCI, and went on to demonstrate an improvement from a complete to an incomplete injury in 5.6% of those subjects [33].

In the present study we enrolled subjects with chronic and complete SCI (ASIA grade A) who had previously been subjected to decompressive surgery and lengthy rehabilitation protocols without acquiring significant motor or sensory gains. Locomotor training has been shown to improve the recovery of walking in many subjects with incomplete SCI, but not in subjects with severe injury [34].

Our primary outcome was safety, and we described adverse events in only one subject who presented a cerebrospinal fluid leak as a postoperative complication not related to the MSCs but rather to the surgical procedure. Cerebrospinal fluid leak is not a routine complication, but may occur in 9% of open spine surgeries [35]. The leak is considered a minor complication because usually it does not change the outcome of the surgery.

Although this was not a controlled study, based on the subject profiles and the expected spontaneous gains, our study showed potential benefits of MSC transplantation treatment in variable degrees of motor and sensory improvements, clinical pain measures and urodynamic parameters. Importantly, we showed that MSC transplantation resulted in the conversion from complete to incomplete injury in seven subjects (58.3%), which was accompanied by improvement in AIS score to grade B or C.

Results obtained in other clinical trials using MSCs for SCI were not as encouraging as those demonstrated here. A study performed with 67 subjects receiving autologous MSC injections intralesionally and intrathecally reported improvements only in one subject, which was considered to be part of the natural history of the disease [23]. A study by Park and colleagues showed significant improvement in three of 10 subjects submitted to intramedullary injection, followed by intrathecal administration of MSCs [24]. Another study evaluated the potential of MSCs injected intrathecally to enhance rehabilitation in 63 subjects with chronic SCI. In the MSC group (consisting of 40 grade A subjects), 12 subjects improved their AIS score of grade A to grade B or C (a conversion rate of 30%), with no statistically significant difference when compared with the control group [36]. Karamouzian and coworkers also tested the safety and feasibility of MSC transplantation by lumbar puncture in 11 subjects, in a nonrandomized clinical trial, comparing the results with those of 20 control subjects who received conventional treatment. Although five of 11 subjects (45.5%) in the study group and three in the control group (15%) demonstrated improvements, there was no statistical significance between the two groups [25].

Variability of the results may be caused by the different numbers of cells, routes and regimen of administration. These are considered key factors for the development of optimized cell-based therapies [37]. The dose and administration routes applied in the present study were established in previous preclinical studies conducted by our group [38, 39]. Based on these preclinical studies, the present trial was the only one to adjust the number of cells administered into the spinal cord to the injury size estimated by MRI. Moreover, considering the studies that used intraspinal injection for MSC delivery, in the present study the number of MSCs was considerably higher than the others, ranging from 4 × 10^6^ to 5 × 10^7^ (median: 2 × 10^7^) versus 3× 10^6^ to 8 × 10^6^[23] and 8 × 10^6^[24].

A recent study evaluated the outcome of 399 subjects with complete thoracic SCI and described a correlation between lower SCI level (T10 to T12) and improved motor function [40]. Although the number of subjects evaluated in our study was less than the aforementioned investigation, we did observe a direct correlation between motor gain and injury level in SCI subjects that received MSC transplantation. Additionally, we found an inverse correlation between light touch gain 6 months after transplantation and volume of the lesion. These results suggest that characteristics of the spinal cord lesion influence the efficacy of the cell therapy, which has not been described before.

Regarding the urodynamic study, we demonstrated a significant enhancement in bladder compliance, which may be an early indication of future improvements in urinary function. Another urodynamic parameter that improved in one-half of the subjects was bladder sensation, which is critical for the subject to feel the optimal moment to initiate bladder emptying. Improvements in bladder sensation avoid elevated filling pressures, which are deleterious to the upper urinary tract. Improvement in sense of bladder filling was seen in only one out of 13 subjects enrolled in a previous study that evaluated the effects of autologous MSC in subjects with chronic SCI [23].

The cell therapy protocol administered in this study was feasible and an overall safe procedure, which capitalized on the use of an autologous and easily obtainable source of cells. Importantly, our data corroborate those obtained in previous studies, in which MSCs administered directly into the injured spinal cord were also considered safe, even when combined with additional MSC administrations applied by lumbar puncture [23, 24].

To rehabilitate subjects bearing complete SCI for long periods, such as those enrolled in our study, is a challenging task. The loss of motor function control as a result of interrupted pathways within the spinal cord after injury has severe consequences for movement recovery, which includes muscle atrophy, joint instability and bone weakness. This suggests that an autologous cell therapy approach, applied as early as possible, may be vital to minimize the development of the deleterious secondary outcomes following SCI and lead to an increase in likelihood of motor recovery. Moreover, the combination of a cell-based therapy with other known factors that potentiate the plasticity of the central nervous system may increase functional recovery [32]. Additionally, the level of measurable benefits that can be achieved may depend on several factors, including the number of MSCs injected into the lesion site and doses, as well as therapeutic window, all of which need to be further investigated in order to optimize the effects of MSC-based therapy for SCI.

## Conclusions

The present study has demonstrated the safety, feasibility and potential efficacy of autologous MSC administration into subjects with chronic SCI. Moreover, injury characteristics such as level and size were found to influence the outcome of cell therapy for SCI. The results indicate potential benefits provided by MSC therapy, which should be confirmed in larger and controlled clinical trials.

## Abbreviations

AIS: American Spinal Injury Association impairment scale
ASIA: American Spinal Injury Association
MRI: magnetic resonance imaging
MSC: mesenchymal stem cell
SCI: spinal cord injury
SSEP: somatosensory evoked potentials
VAS: visual analogue score.
